# Association between smoking status and the parameters of vascular structure and function in adults: results from the EVIDENT study

**DOI:** 10.1186/1471-2261-13-109

**Published:** 2013-12-01

**Authors:** Jose I Recio-Rodriguez, Manuel A Gomez-Marcos, Maria C Patino Alonso, Carlos Martin-Cantera, Elisa Ibañez-Jalon, Amor Melguizo-Bejar, Luis Garcia-Ortiz

**Affiliations:** 1 La Alamedilla Health Centre, Castilla y León Health Service–SACYL, redIAPP, IBSAL, Salamanca, Spain; 2 Statistics Department, University of Salamanca, Salamanca, Spain; 3 Passeig de Sant Joan Health Centre, Catalan Health Service, Barcelona, Spain; 4 Casa de Barco Health Centre, Castilla y León Health Service–SACYL, Valladolid, Spain; 5 Torre Ramona Health Centre, Aragón Health Service, Zaragoza, Spain

**Keywords:** Smoking, Carotid intima-media thickness, Vascular stiffness

## Abstract

**Background:**

The present study analyses the relation between smoking status and the parameters used to assess vascular structure and function.

**Methods:**

This cross-sectional, multi-centre study involved a random sample of 1553 participants from the EVIDENT study. Measurements: The smoking status, peripheral augmentation index and ankle-brachial index were measured in all participants. In a small subset of the main population (265 participants), the carotid intima-media thickness and pulse wave velocity were also measured.

**Results:**

After controlling for the effect of age, sex and other risk factors, present smokers have higher values of carotid intima-media thickness (p = 0.011). Along the same lines, current smokers have higher values of pulse wave velocity and lower mean values of ankle-brachial index but without statistical significance in both cases.

**Conclusions:**

Among the parameters of vascular structure and function analysed, only the IMT shows association with the smoking status, after adjusting for confounders.

## Background

A consistent relationship has been demonstrated between cigarette smoke exposure and the progression of carotid atherosclerosis [1], with a strong positive association with coronary artery calcium burden [2]. Smoking has been associated with increased arterial stiffness and central hemodynamic indices [3-6]. There is evidence that the ankle-brachial index inversely and linearly correlates with cigarette smoking [7,8]. Nevertheless, when evaluating vascular structure and function, every test has different accessibility and costs [9]. Several authors have proposed that the patient’s age, sex, blood pressure and heart rate, and the presence of obesity, diabetes and vascular drugs, are the main determinants of the parameters that assess arterial stiffness and vascular function [10-13]. The aim of this study was to assess the relationship between smoking status and vascular structure and function in a random sample of the adult population from the EVIDENT study.

## Methods

### Study design and population

The EVIDENT study is a cross-sectional and multi-centre study of six patient groups distributed throughout Spain. Participants, aged 20–80 years, were selected by stratified random sampling. The following exclusion criteria were applied: known coronary or cerebrovascular atherosclerotic disease, heart failure, moderate or severe chronic obstructive pulmonary disease, walking-limiting musculoskeletal disease, advanced respiratory, renal or hepatic disease, severe mental disease, treated oncological disease diagnosed in the past 5 years and terminal illness. The study was approved by an independent ethics committee from Salamanca University Hospital (Spain), and all participants provided written informed consent according to the general recommendations of the Declaration of Helsinki. The recruitment and data collection were conducted between 2011 and 2012. A total of 1553 individuals were included in the study. The sample size calculation indicated that this number was sufficient to detect a difference of 5 units in the peripheral augmentation index between 3 smoking statuses (i.e., smoker, former smoker and non-smoker) in a two-sided test, assuming a common standard deviation (SD) of 21 units with a significance level of 95% and a power of 90%. The IMT and PWV were measured in only 265 participants, but this number was sufficient to detect a 0.05 mm difference in the IMT between the 3 groups, assuming a SD of 0.1, a significance level of 95% and a power of 80%. The findings presented in this manuscript are a subanalysis of the EVIDENT study, the main results of which were recently published [14].

### Variables and measurement instruments

Smoking history was assessed by asking questions about the participant’s smoking status. For the analyses, the participants were classified as non-smokers, former (>1 year without smoking) or present smokers. Carotid ultrasonography to assess intima-media thickness of the common carotid artery (C-IMT) was performed with the Sonosite Micromax ultrasound device (Sonosite Inc., Bothell, Washington, USA) paired with a 5–10 MHz multi-frequency high-resolution linear transducer. Sonocal software was used to perform automatic IMT measurements. Six measurements were performed on each carotid artery using average values (average IMT) and maximum values (maximum IMT) automatically calculated by the software. The measurements were taken following the recommendations of the Manheim Carotid Intima-Media Thickness Consensus [15]. Carotid-femoral pulse wave velocity (PWV) was estimated using the SphygmoCor System (AtCor Medical Pty Ltd., Head Office, West Ryde, Australia), according to the expert consensus document on arterial stiffness by Van Bortel et al. [16]. The central blood pressure and radial or peripheral augmentation index (PAIx) were measured with the Pulse Wave Application Software (A Pulse) (HealthSTATS International, Singapore) using tonometry to capture the radial pulse and to estimate the central blood pressure using a patented equation. The PAIx was calculated as follows: (second peak systolic blood pressure [SBP2] - diastolic blood pressure [DBP])/ (first peak SBP - DBP) × 100 (%). The PAIx was standardised to a heart rate of 75 bpm. The ankle-brachial index (ABI) was measured using a portable WatchBP Office ABI (Microlife AG Swiss Corporation). The ABI was calculated automatically dividing the higher of the two ankle systolic pressures by the highest measurement of the two systolic pressures in the arm [17]. All measurements (IMT, PWV, PAIx75 and ABI) were performed in the morning. Smoking was not allowed within the 3 h prior to the measurements. Further details on the EVIDENT study design have been published elsewhere [18].

### Statistical analysis

Statistical normality was checked using the Kolmogorov–Smirnov test. Normally distributed continuous variables were expressed as the mean ± standard deviation, while non-normally distributed variables were presented as median and 75–25th percentile. Frequency distribution was used for the categorical variables. The difference in means in continuous variables between the smoking categories was analysed using a one-way analysis of variance for independent samples and the post-hoc Scheffé contrast, with alpha <0.05 and the Kruskal–Wallis test when the variables were not normal. Chi-squared tests were used to compare the differences in categorical variables.

Age, sex, blood pressure, heart rate, and the presence of obesity, diabetes and vascular drugs have shown to affect the PWV, IMT or augmentation index values. Therefore, it is necessary to control the effect of these variables in the relationship between smoking status and the parameters of vascular structure and function. To analyse the relationship between the vascular structure and function (IMT, PAIx75, PWV or ABI) and smoking status (non-smoker = 0, present smoker = 1, former smoker =2), a general linear model (GLM) analysis was performed, including age, sex, systolic blood pressure, heart rate, body mass index, HDL cholesterol, diabetes and the presence of antihypertensive, antidiabetic and lipid-lowering drugs as the adjustment variables. The non-normal variables were modelled as continuous variables with log transformation to achieve normality in the multivariate analysis. Data were analysed using the SPSS version 18.0 statistical package (SPSS Inc., Chicago, Illinois, USA), and p < 0.05 was considered to be statistically significant.

## Results

Table 1 shows the clinic characteristics of each group according to its smoking status. Current smokers are younger and have the lowest prevalence of hypertension, obesity and dyslipidemia. PWV, IMT and ABI are lower in present smokers, while PAIx75 are higher in these individuals. An analysis of the 265 individuals for whom the IMT and PWV were performed is shown in Additional file 1: Table S1. The results showed that the demographic and biological characteristics, according to smoking status, were similar to the overall analysed sample. The mean package year in the present smokers was 16.78 ± 16.31, and the average smoking history was 30.39 ± 12.57 years. In an age-adjusted correlation, we found a positive correlation between the package years and the PAIx75 (r = 0.332, p = 0.015) and a negative correlation with the ABI (r = -289; p = 0.036). The PWV and IMT showed no significant correlations.

**Table 1:** Characteristics of patients by smoking status

	**Nonsmokers (n = 747)**	**Former smokers (n = 469)**	**Present smokers (n = 337)**	**p value**
Age (years)*¥	56.14 (65.09-43.64)	55.69 (64.44-46.71)	48.24 (56.08-37.25)	<0.001
Males (%)	212 (28.4)	271 (57.8)	133 (39.5)	<0.001
Hypertension (%)	307 (41.1)	219 (46.7)	103 (30.6)	<0.001
Diabetes (%)	79 (10.6)	65 (13.9)	34 (10.1)	0.145
Dyslipidemia (%)	214 (28.6)	177 (37.7)	82 (24.3)	<0.001
Obesity (%)	161 (21.6)	114 (24.3)	62 (18.4)	0.124
Antihypertensive drugs (%)	223 (29.9)	162 (34.5)	66 (19.6)	<0.001
Lipid-lowering drugs (%)	126 (16.9)	122 (26.0)	48 (14.2)	<0.001
Antidiabetic drugs (%)	52 (7.0)	46 (9.8)	25 (7.4)	0.187
Office SBP (mmHg)*¥	123.75 (134.63-113.50)	125.50 (138.00-114.50)	121.25 (131.38-109.13)	<0.001
Office DBP (mmHg)	76.50 (83.50-70.50)	77.00 (83.38-70.00)	75.50 (82.50-70.00)	0.377
Office heart rate (bpm)*¥#	71.50 (78.50-64.50)	69.50 (76.50-61.13)	75.00 (82.63-67.00)	<0.001
Central SBP (mmHg)*¥	124 (135–114)	125 (137–115)	121 (132–110)	0.002
Central DBP (mmHg)	76 (83–70)	77 (84–70)	75 (82–68)	0.188
BMI (kg/m^2^)*¥	26.67 (29.56-24.05)	27.07 (29.96-24.90)	25.64 (28.52-22.90)	<0.001
Waist circumference (cm) ¥#	92 (100–84)	95 (104–88)	91.00 (99–82)	<0.001
Total cholesterol (mg/dL)	215.41 ± 38.77	213.36 ± 39.10	210.74 ± 39.28	0.192
Triglycerides (mg/dL)*	95 (133–69)	96 (136–73)	104 (147–75)	0.026
LDL-cholesterol (mg/dL)	133.85 ± 36.78	133.18 ± 34.89	132.36 ± 36.75	0.827
HDL-cholesterol (mg/dL)*#	59.00 (70.00-49.00)	56.00 (67.00-47.00)	54.00 (64.75-45.00)	<0.001
Mean IMT (mm) (n = 265) ¥	0.68 ± 0.10	0.70 ± 0.11	0.65 ± 0.11	0.045
Maximum IMT (mm) (n = 265) ¥	0.83 ± 0.12	0.86 ± 0.13	0.81 ± 0.13	0.042
PWV (m/sec) (n = 265)	7.10 (8.60-6.38)	7.30 (8.95-6.35)	6.65 (7.95-5.57)	0.032
PAIx75 (%) #¥	89 (101–78)	86 (97–75)	90 (103–77)	0.003
ABI #¥	1.17 ± 0.13	1.19 ± 0.14	1.16 ± 0.14	0.005

Normally distributed continuous variables are expressed as mean ± standard deviation, while non-normally distributed variables are presented as median and 75–25th percentile. Frequency distribution was used in categorical variables.

Obesity: *BMI* ≥ 30 Kg/m^2^or Waist circumference ≥ 88 cm in women and ≥ 102 cm in men. *SBP*: Systolic blood pressure; *DBP*: Diastolic blood pressure; *BMI*: body mass index; *HDL*: high density lipoprotein; *LDL*: lowdensity lipoprotein; *IMT*: Intima Media Thickness; *PWV*: pulse wave velocity; *PAIx*75: Peripheral or radial augmentation index adjusted for heart rate at 75 bpm, *ABI*: ankle brachial index.

*Differences between present and nonsmokers, ¥: Differences between present and former smokers, #: Differences between former and nonsmokers.

Table 2 shows the IMT, PWV, PAIx75 and ABI values according to patient sex; the presence of diabetes, antihypertensive, antidiabetic and lipid-lowering drugs. IMT and PWV are higher in the male participants, diabetics and patients undergoing vascular treatment. The PAIx 75 shows the highest values in females, individuals with diabetes and patients undergoing vascular treatments, while the ABI shows differences in individuals with and without diabetes.

**Table 2:** **Values of vascular structure and functional parameters according to sex and presence of diabetes**, **antihypertensive**, **antidiabetic and lipid lowering drugs**

	**PWV (n = 265)**		**IMT (n = 265)**		**PAIx75 (n = 1553)**		**ABI (n = 1553)**	
		**p-value**		**p-value**		**p-value**		**p-value**
Sex								
Male	7.6 (6.7-9.5)	<0.001	0.71 ± 0.12	<0.001	83 (94–71)	<0.001	1.18 ± 0.14	0.455
Female	6.8 (6.0-7.9)		0.66 ± 0.09		91 (105–81)		1.17 ± 0.13	
Diabetes								
Yes	9.6 (8.3-10.9)	<0.001	0.74 ± 0.07	0.007	93 (104–92)	0.001	1.15 ± 0.16	0.022
No	7.0 (6.1-8.2)		0.67 ± 0.11		87 (100–77)		1.18 ± 0.13	
Antihypertensive drugs								
Yes	8.3 (7.3-9.8)	<0.001	0.74 ± 0.10	<0.001	89 (102–79)	0.033	1.18 ± 0.14	0.693
No	6.8 (5.9-7.8)		0.66 ± 0.10		87(100–76)		1.17 ± 0.13	
Antidiabetic drugs								
Yes	9.5 (10.6-8.5)	<0.001	0.74 ± 0.08	0.048	93 (102–83)	0.004	1.14 ± 0.16	0.007
No	7.0 (8.2-6.2)		0.68 ± 0.10		88 (100–77)		1.18 ± 0.13	
Lipid lowering drugs								
Yes	8.1 (6.9-10.8)	<0.001	0.74 ± 0.09	<0.001	91 (103–80)	0.004	1.17 ± 0.14	0.438
No	6.9 (6.1-8.2)		0.67 ± 0.10		87 (100–77)		1.17 ± 0.13	

*IMT*: Intima Media Thickness; *PWV*: pulse wave velocity; *PAIx*75: Peripheral or radial augmentation index adjusted for heart rate at 75 bpm, *ABI*: ankle brachial index.

*PWV* and PAIx75 are showed in median and interquartil range and *IMT* and *ABI* as mean ± Standard deviation.

p-values are for comparison of subgroups by T-Student independent groups and U Mann Whitney test.

**Figure 1:**
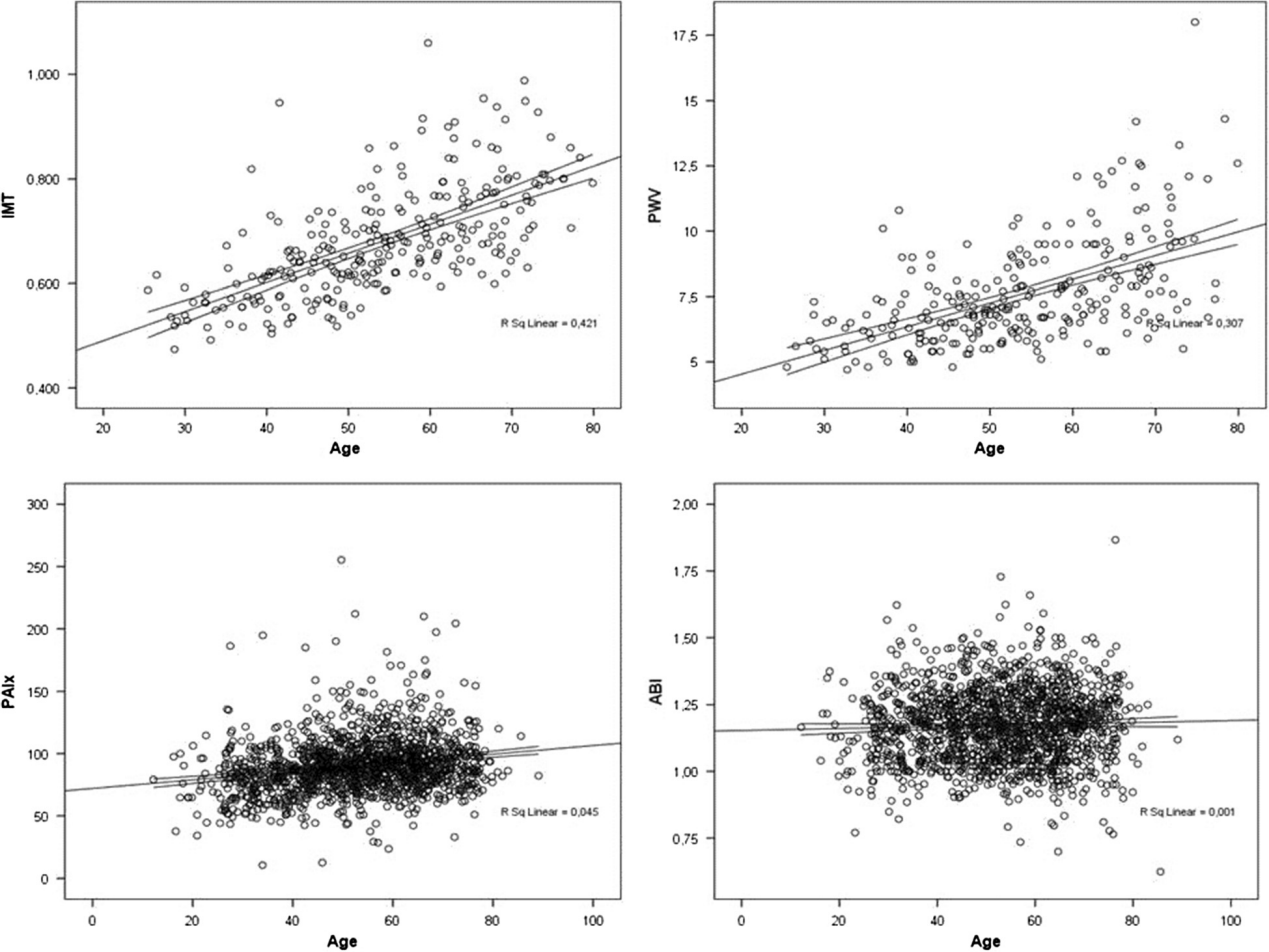
Scatterplots representing the relationship between age and each vascular structure and functional parameter analysed (PWV r = 0.558; p < 0.01, IMT r = 0.687; p < 0.01, PAIx75 r = 0.222; p < 0.01 and ABI r = 0.045; p > 0.05).

Table 3 shows a bivariate correlation between systolic blood pressure, heart rate and body mass index with each vascular structure and functional parameter (i.e., PWV, IMT, PAIx75 and ABI) analysed. Age shows a linear relationship with PWV, IMT and PAIx75 (Figure 1).

**Table 3:** **Bivariate correlations between vascular structure and functional parameters and systolic blood pressure**, **heart ratio and body mass index**

	**Age**	**SBP**	**HR**	**BMI**
PWV (n = 265)	0.558**	0.623**	0.169**	0.353**
IMT (n = 265)	0.687**	0.441**	-0.054	0.250**
PAIx75 (n = 1553)	0.222**	0.134**	0.361**	0.103**
ABI (n = 1553)	0.045	-0.063*	-0.038	0.033

*p < 0.05, **p < 0.01.

*SBP*: Systolic blood pressure; *HR*: heart rate; *BMI*: body mass index; *IMT*: Intima Media Thickness; *PWV*: pulse wave velocity; *PAIx*75: Peripheral or radial augmentation index adjusted for heart rate at 75 bpm, *ABI*: ankle brachial index.

**Figure 2:**
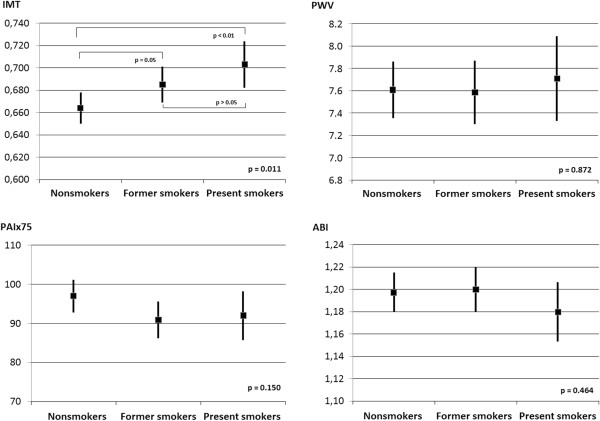
**Relationship between the smoking status, vascular structure and function parameters adjusted by age, sex, systolic blood pressure, heart rate, body mass index, HDL cholesterol, diabetes and the presence of antihypertensive, antidiabetic and lipid-lowering drugs.** Figure represents the adjusted means and 95% CI. Statistical significance: IMT p = 0.011*, PWV p = 0.872, PAIx75 p = 0.150, ABI p = 0.464.

After controlling for the effects of age, sex, systolic blood pressure, heart rate, body mass index, HDL-cholesterol, diabetes and the presence of antihypertensive, antidiabetic and lipid-lowering drugs, the multivariate analysis shows that present smokers have higher IMT values (p = 0.011). PWV behaves likewise, although it does not reach the level of statistical significance. ABI has no modifications, and PAIx75 has higher values in current smokers than in former smokers (Figure 2). More details of the multivariate analysis are shown in Table 2 of the Additional file 2: (Table S2).

## Discussion

In this paper, we present data about the relationship between smoking status and a large variety of parameters that assess vascular structure and function in a general population sample from primary care clinics. To assess vascular structure and function, parameters differ in their relationships with cardiovascular risk factors. The results of this work show that IMT is the parameter that best relates to smoking status in a representative sample of adult population. After controlling for the effects of sex and other confounders, present smokers have higher mean IMT values (p = 0.011), while the differences in PWV, PAIx75 and ABI did not reach the level of statistical significance.

The study results concerning the effects of smoking status on subclinical arterial disease align with those of other authors [1,2], indicating that smoking relates to the presence of subclinical atherosclerosis in an adult population. Some authors have reported that smoking aligned with increased inflammatory markers [19,20]. Other authors have shown that the polymorphism -930A/G may modify the association between smoking and IMT values, particularly among healthy young adults [21]. We found no correlation between the mean package years in present smokers with IMT. This result can be explained by the small number of present smokers for whom the IMT was analysed.

After controlling for confounders, we found no association between the presence of smoking and increased arterial stiffness. In a systematic literature review, Doonan RJ et al. [22] found that some studies found no significant difference in the arterial stiffness between non-smokers and long-term smokers; they concluded that the effect of smoking on arterial stiffness remains to be established by prospective smoking cessation trials. Rhee et al. found an association between PWV and cigarette smoking in male smokers with hypertension. Their study explored the acute effects of smoking in a sample of men with and without hypertension, while our work examined the chronic effects of smoking on a larger sample of the Spanish general population. Other differences between the study of Rhee and Kubozono [3,4] and our work are the different variables used in the multiple regression models. Age and SBP [10] are among the major determinants of PWV. In our study, the smoker group was the youngest and had lower SBP values. Although these variables were included in the multivariate analysis model, it may not be sufficient to control the effects that they may have on the study results.

Previous studies have demonstrated an association between AIx and cardiovascular risk factors, including smoking [6,23]. In our work, the highest BMI corresponds to the former smokers group, which could explain the lower value of PAIx75 in this group because the augmentation index decreases when BMI increases [24]. Among the major determinants of PAIx75 are the patient age, SBP and BMI. In our study, the smoking group had the youngest participants and lower SBP and BMI levels. Although these two variables were included in the multivariate analysis, they may not sufficiently counteract the effects of other variables in the study. Furthermore, Janner JH et al. and Minami J et al. [6,23] analysed the relationship of smoking with CAIx, while in our work we use the radial or peripheral augmentation index. Radial and central AIx are not interchangeable in the clinical practice, although the radial augmentation index has been established as a marker of vascular aging [25].

Present smokers have lower ABI values, similar to the results of other authors [7,26]. However, our results did not remain significant after adjusting for confounders. The study population of Lee YH et al. [26] was from the general population with a mean age of 65 and 70 years in subjects with and without peripheral arterial disease, respectively. The peripheral arterial disease is one of the major manifestations of generalised atherosclerotic disease, as a result of progressive atherosclerosis [27]; therefore, it is expected that individuals with longer smoking histories will have lower ABI values. The population studied in our work has a lower median age (52.9 ± 13 years), with the youngest members in the smoking group.

The main limitation of this study is the cross-sectional design that prevents the establishment of causal relationships between smoking and vascular structure and function. The participants of the three smoking categories differed in terms of age and the prevalence of other risk factors. This limitation is, to some extent, addressed by the statistical analysis that controls the effect of these variables in the interpretation of results. Another limitation of this study is that the smoking status was self-reported and not determined by objective measures, such as CO in the expired air analysis; however, such questionnaires have been used previously in other studies to explore the relationship between arterial stiffness and smoking [5]. Lastly, a full set of data (IMT and PWV) are only available in a small subset of the EVIDENT study. However, this sample has similar demographic and biological characteristics, according to smoking status, compared to the overall sample analysed.

## Conclusions

Among the parameters vascular structure and function analysed, only the IMT shows association with the smoking status, after adjusting for confounders. Further studies focusing on the smoking statuses of participants are necessary to clarify the role of the chronic effects of smoking on the parameters of vascular structure and function and the effects of passive smoking exposure.

## Abbreviations

BMI: Body mass index; PAIx75: Peripheral or radial augmentation index adjusted for heart rate at 75 bpm; ABI: Ankle brachial index; IMT: Intima-media thickness of the common carotid artery; PWV: Pulse wave velocity.

## Competing interests

The author’s declare that they have no competing interests.

## Authors’ contribution

JIR devised the study, designed the protocol, assisted with fund raising and results interpretation, prepared the draft of the manuscript and corrected the final version of the manuscript. MAG, CM, EI and AM participated in the study design, results interpretation, and manuscript review. CA participated in the study design, data collection and manuscript review. MCP performed all analytical methods, results interpretation, and manuscript review. LG participated in the protocol design, fund raising, analysis of results, and final review of the manuscript. Finally, all authors reviewed and approved the final version of the manuscript.

## Pre-publication history

The pre-publication history for this paper can be accessed here:

http://www.biomedcentral.com/1471-2261/13/109/prepub

## Supplementary Material

Additional file 1: Table S1 Characteristics of patients by smoking status in the 265 subjects for whom the IMT and the PWV was assessed. Click here for file

Additional file 2: Table S2 Multivariate analysis of structure and function vascular parameters with smoking status (GLM). Click here for file

## EVIDENT group members

Coordinating centre: Luis Garcia Ortiz, Manuel A Gómez Marcos, José I Recio Rodriguez and Carmen Patino Alonso of the Primary Care Research Unit of La Alamedilla Health Centre, Salamanca, Spain.

Participating centres: La Alamedilla Health Center, Servicio de Salud de Castilla y León SACYL: Carmen Castaño Sánchez, Carmela Rodriguez Martín, Yolanda Castaño Sánchez, Cristina Agudo Conde, Emiliano Rodriguez Sánchez, Luis J Gonzalez Elena, Carmen Herrero Rodriguez, Benigna Sánchez Salgado, Angela de Cabo Laso and Jose A Maderuelo Fernández.

Passeig de Sant Joan Health Centre, Servicio de Salud Catalan: Carlos Martín Cantera, Joan Canales Reina, Epifania Rodrigo de Pablo, Maria Lourdes Lasaosa Medina, Maria Jose Calvo Aponte, Amalia Rodriguez Franco, Elena Briones Carrio, Carme Martin Borras, Anna Puig Ribera and Ruben Colominas Garrido.

Poble Sec Health Centre, Servicio de Salud Catalan: Juanjo Anton Alvarez, Mª Teresa Vidal Sarmiento, Ángela Viaplana Serra, Susanna Bermúdez Chillida, Aida Tanasa.

Ca N’Oriac Health Centre, Servicio de Salud Catalan: Montserrat Romaguera Bosch.

Sant Roc Health Centre, Servicio de Salud Catalan: Maria Mar Domingo, Anna Girona, Nuria Curos, Francisco Javier Mezquiriz, Laura Torrent.

Cuenca III Health Centre, Servicio de Salud de Castilla-La Mancha SESCAM: Alfredo Cabrejas Sánchez, María Teresa Pérez Rodríguez, María Luz García García, Jorge Lema Bartolomé and Fernando Salcedo Aguilar.

Casa de Barco Health Centre, Servicio de Salud de Castilla y León SACYL: Carmen Fernandez Alonso, Amparo Gómez Arranz, Elisa Ibáñez Jalón, Aventina de la Cal de la Fuente, Laura Muñoz Beneitez, Natalia Gutiérrez, Ruperto Sanz Cantalapiedra, Luis M Quintero Gonzalez, Sara de Francisco Velasco, Miguel Angel Diez Garcia, Eva Sierra Quintana and Maria Cáceres.

Torre Ramona Health Centre, Servicio de Salud de Aragon: Natividad González Viejo, José Felix Magdalena Belio, Luis Otegui Ilarduya, Francisco Javier Rubio Galán, Amor Melguizo Bejar, Cristina Inés Sauras Yera, Mª Jesus Gil Train, Marta Iribarne Ferrer and Miguel Angel Lafuente Ripolles.

Primary Care Research Unit of Bizkaia, Basque Health Service-Osakidetza: Gonzalo Grandes, Alvaro Sanchez, Nahia Guenaga, Veronica Arce, Maria SoledadArietaleanizbealoa, Eguskiñe Iturregui San Nicolás, Rosa Amaia Martín Santidrián and Ana Zuazagoitia.
