# Supplementary material for: Association between smoking status and the parameters of vascular structure and function in adults: results from the EVIDENT study
Source: BMC Cardiovasc Disord. 2013 Dec 1;13:109. doi: 10.1186/1471-2261-13-109 (PMC4219389; doi:10.1186/1471-2261-13-109)
Supplement: Additional file 1: Table S1 — Characteristics of patients by smoking status in the 265 subjects for whom the IMT and the PWV was assessed. [file 1471-2261-13-109-S1.doc]

**Additional file 1: Table S1** Characteristics of patients by smoking status in the 265 subjects for whom the IMT and the PWV was assessed.

|  | Nonsmokers (n=120) | Former smokers (n=91) | Present smokers (n=54) | p value |
| --- | --- | --- | --- | --- |
|  |  |  |  |  |
| Age (years) | 56.67±12.14 | 54.86±11.42 | 46.11±10.24 | <0.001 |
| Males (%) | 33 (27.5) | 52 (57.1) | 23 (42.6) | <0.001 |
| Hypertension (%) | 39 (32.5) | 31 (34.1) | 6 (11.1) | 0.006 |
| Diabetes (%) | 4 (3.3) | 10 (11.0) | 3 (5.6) | 0.077 |
| Dyslipidemia (%) | 38 (31.7) | 27 (29.7) | 9 (16.7) | 0.112 |
| Obesity (%) | 26 (21.7) | 24 (26.4) | 6 (11.1) | 0.092 |
| Antihypertensive Drugs (%) | 38 (31.7) | 33 (36.3) | 6 (11.1) | 0.004 |
| Lipid-lowering Drugs (%) | 23 (19.2) | 19 (20.9) | 5 (9.3) | 0.179 |
| Antidiabetic Drugs (%) | 4 (3.3) | 6 (6.6) | 2 (3.7) | 0.502 |
| Office SBP (mmHg) | 122.83±16.45 | 124.11±18.19 | 118.11±19.67 | 0.133 |
| Office DBP (mmHg) | 77.70±9.93 | 78.35±11.31 | 76.09±11.85 | 0.476 |
| Office heart rate (bpm) | 69.49±11.24 | 66.96±10.51 | 69.89±9.60 | 0.157 |
| Central SBP (mmHg) | 115.18±15.34 | 115.61±18.25 | 111.39±16.60 | 0.291 |
| Central DBP (mmHg) | 76.92±10.81 | 77.22±15.60 | 74.28±12.58 | 0.371 |
| BMI (kg/m^2^) | 27.37±4.33 | 28.19±3.93 | 26.15±5.50 | 0.031 |
| Waist circumference (cm) | 92.58±9.75 | 96.58±11.69 | 91.37±14.15 | 0.011 |
| Total cholesterol (mg/dL) | 219.81±38.97 | 209.25±39.15 | 200.15±30.90 | 0.004 |
| Triglycerides (mg/dL) | 103.65±47.99 | 114.55±62.83 | 117.09±109.65 | 0.378 |
| LDL-cholesterol (mg/dL) | 136.95±32.12 | 130.68±36.65 | 122.88±29.04 | 0.038 |
| HDL-cholesterol (mg/dL) | 62.60±16.43 | 55.71±15.00 | 54.11±12.91 | <0.001 |
| Mean IMT (mm) | 0.68±0.10 | 0.70±0.11 | 0.65±0.11 | 0.045 |
| PWV (m/sec) | 7.69±1.91 | 7.83±2.22 | 7.02±1.73 | 0.053 |
| PAIx75 (%) | 99.76±25.97 | 88.75±23.66 | 88.18±16.31 | <0.001 |
| ABI | 1.20±0.10 | 1.21±0.08 | 1.18±0.11 | 0.173 |

Normally distributed continuous variables are expressed as mean ± standard deviation, while non-normally distributed variables are presented as median and 75–25th percentile. Frequency distribution was used in categorical variables.

Obesity: BMI ≥ 30 Kg/m²or Waist circumference ≥ 88 cm in women and ≥ 102 cm in men. SBP: Systolic blood pressure; DBP: Diastolic blood pressure; BMI: body mass index; HDL: high density lipoprotein; LDL: lowdensity lipoprotein; IMT: Intima Media Thickness; PWV: pulse wave velocity; PAIx75: Peripheral or radial augmentation index adjusted for heart rate at 75 bpm, ABI: ankle brachial index.
