# Supplementary material for: Association between smoking status and the parameters of vascular structure and function in adults: results from the EVIDENT study
Source: BMC Cardiovasc Disord. 2013 Dec 1;13:109. doi: 10.1186/1471-2261-13-109 (PMC4219389; doi:10.1186/1471-2261-13-109)
Supplement: Additional file 2: Table S2 — Multivariate analysis of structure and function vascular parameters with smoking status (GLM). [file 1471-2261-13-109-S2.doc]

**Additional file 2: Table S2 Multivariate analysis of structure and function vascular parameters with smoking status (GLM)**

| **Dependent variable** | **Independent variable** | **Mean** | **Standard error.** | **CI 95%** | | **p value** |
| --- | --- | --- | --- | --- | --- | --- |
| IMT | Nonsmokers | 0,66 | 0,01 | 0,65 | 0,68 | 0,011 |
|  | Former smokers | 0,69 | 0,01 | 0,67 | 0,70 |  |
|  | Present smokers | 0,70 | 0,01 | 0,68 | 0,72 |  |
| PWV | Nonsmokers | 7,61 | 0,13 | 7,35 | 7,86 | 0,872 |
|  | Former smokers | 7,58 | 0,14 | 7,30 | 7,87 |  |
|  | Present smokers | 7,71 | 0,19 | 7,33 | 8,09 |  |
| PAIx75 | Nonsmokers | 96,97 | 2,12 | 92,80 | 101,15 | 0,150 |
|  | Former smokers | 90,89 | 2,38 | 86,21 | 95,57 |  |
|  | Present smokers | 91,98 | 3,18 | 85,72 | 98,24 |  |
| ABI | Nonsmokers | 1,20 | 0,01 | 1,18 | 1,22 | 0,464 |
|  | Former smokers | 1,20 | 0,01 | 1,18 | 1,22 |  |
|  | Present smokers | 1,18 | 0,01 | 1,15 | 1,21 |  |

Adjusted for age, sex, systolic blood pressure, body mass index, HDL cholesterol, diabetes and the presence of antihypertensive, antidiabetic and lipid-lowering drugs
